# Better detection of reduced motor functioning in brain tumor survivors based on objective motor assessments: an incentive for improved standardized follow-up

**DOI:** 10.1007/s00431-022-04472-1

**Published:** 2022-04-27

**Authors:** Marjoke Gielis, Veerle Dirix, Ellen Vanderhenst, Anne Uyttebroeck, Hilde Feys, Charlotte Sleurs, Sandra Jacobs

**Affiliations:** 1 Department of Pediatrics, Pediatric Hemato-Oncology, University Hospitals Leuven, Herestraat 49, Leuven, Belgium; 2 Department of Oncology, KU Leuven, Leuven, Belgium; 3 Department of Rehabilitation Sciences, KU Leuven, Leuven, Belgium

**Keywords:** Pediatric, Brain tumor, Follow-up, Objective motor functioning, Questionnaire

## Abstract

Long-term sequelae are well-known in childhood brain tumor survivors, but motor functioning remains poorly described. This cross-sectional study aimed to assess objective motor functioning, patient-specific risk factors, and parental perceptions. Fifty-two childhood brain tumor patients (pilocytic astrocytoma, medulloblastoma, and other types) who were at least 6 months out of treatment were evaluated. Mean age at testing was 11.7 years. Objective motor functioning was assessed with the Movement Assessment Battery for Children (MABC-2-NL) and/or Bruininks-Oseretsky test of motor proficiency (BOT-2). Functional walking capacity was assessed with the 6-min walk test (6MWT). Parent-reported motor functioning was addressed using the ABILHAND-Kids, ABILOCO-Kids questionnaires, and a standardized anamnesis. Patients showed impaired motor functioning in all domains (*p* < 0.001). Regarding risk factors, younger age at diagnosis (< 5 year) was significantly associated with lower scores on body coordination (*p* = 0.006). Adjuvant treatment resulted in lower scores for fine manual control of the BOT-2 (*p* = 0.024) and balance of MABC-2-NL (*p* = 0.036). Finally, questionnaires revealed an underestimation of motor problems as perceived by the parents. In conclusion, many children who are in follow-up for a brain tumor show impaired motor functioning on multiple aspects, with younger age at diagnosis and adjuvant treatment as specific risk factors. Based on the questionnaires and anamnesis, motor problems appear to be underestimated by the parents.

*  Conclusion*: These findings point to the need for timely prospective screening of motor functioning. Based on a screening assessment, adequate rehabilitation programs can be applied in childhood brain tumor survivors, aiming to reduce the adverse impact on their daily lives, both for functional activities and cardiovascular fitness.

**Table:** 

**What is Known:** • *A pediatric brain tumor and its treatment are associated with potential long-term motor sequelae.* • *Test assessments could enable us to objectify motor functioning of these patients.*	
**What is New:** • *Pediatric brain tumors survivors show lower motor performance compared to the norm, which is often underestimated by parents.* • *Younger age at diagnosis and adjuvant treatment could be specific risk factors.*

**Supplementary information:**

The online version contains supplementary material available at 10.1007/s00431-022-04472-1.

## Introduction

The population of survivors of childhood brain tumors is expanding due to more successful treatments [1]. Consequently, potential adverse long-term effects of the treatment, which typically consists of surgery, radiotherapy, chemotherapy, or a combination of these, increasingly require attention [1]. More specifically, there is an increased risk to develop long-term motor impairment, endocrine disorders, cardiovascular conditions, and cognitive deficits [2–5]. In a study investigating health-related quality of life (HRQL), pediatric brain tumor survivors obtained lower scores in multiple domains, compared to other cancer survivors or healthy peers [6]. In another study, Aarsen et al. indicated physical, motor, autonomic, cognitive, and social functioning and positive emotions to be significantly lower for patients compared to healthy peers [7]. Parents judged the HRQL of their child to be poorer than the children themselves regarding physical, motor, and autonomy domains [16]. Due to chemotherapy, peripheral neuropathy decreases in muscle strength, and damage to the central nervous system can occur, posing these patients at increased risk for reduced motor proficiency [8]. However, studies investigating objective motor functioning in childhood cancer survivors are rather scarce, particularly in childhood brain tumors. Conklin et al. [9] used the Bruininks-Oseretsky test of motor proficiency (BOT-2) to evaluate motor performance of patients with craniopharyngioma during treatment. They concluded that children with craniopharyngioma demonstrated significantly reduced aerobic fitness, motor proficiency, and working memory, which were positively correlated. This association between cardiorespiratory fitness and working memory in childhood brain tumor survivors was also confirmed in the study of Wolfe et al. [10]. This implies that neurocognitive decline, which is abundantly evidenced in this population [11], could be inter-related with existing cardiovascular or motor problems. More specifically, fine motor coordination and speed (assessed with the Purdue and Grooved Pegboard) of pediatric brain tumor patients and survivors was reduced compared to the normative sample [12]. Such fine motor skills depend on both processing speed and coordinating skills.

Piscione et al. [13] performed an investigation of motor functioning post-treatment. Their results revealed significant differences between children with brain tumors and normative data for body coordination and strength and agility using the BOT-2. Varedi et al. [14] observed balance impairments in 48% of adult survivors of pediatric central nervous tumors. Another study evaluated cardiorespiratory fitness with VO_2 max_ testing in pediatric fossa posterior tumor survivors. These children were less fit than healthy peers or children with pulmonary disease but similar to patients with chronic heart disease and other types of childhood cancer [15].

Regarding potential risk factors, Piscione et al. [13] reported vermis infiltration of the tumor as risk factor for lower body coordination scores and chemo- and radiotherapy for lower strength and agility scores, whereas age at diagnosis (< 3 years versus older) and time since diagnosis were not significant risk factors. Varedi et al. [14] reported that balance impairments were associated with infratentorial tumor location, shunt placement, increased body fat percentage, hearing loss, flexibility limitations, peripheral neuropathy, and cognitive deficits.

Although survivors report more physical difficulties at group level, it remains inconclusive which patients are at increased risk [16, 17]. This limited amount of evidence is in contrast to more extensive investigations of risk factors for cognitive functioning (e.g., larger tumors, whole-brain radiation therapy, younger age at diagnosis, and male gender) [18].

More research is required to better map motor deficits in children with both infra- and supratentorial brain tumors. Unlike severe motor impairment, mild post-treatment motor sequelae are often not detected at an early stage. Consequently, adequate rehabilitation in the early phase is regularly lacking. Since the study of Piscione et al. [13] only focused on gross motor skills and on fossa posterior brain tumors, limited information is currently available on fine motor skills and functional capacity.

Within this context, the objectives of this study are (1) to map several components of overall motor function (fine motor function, balance, and coordination) and functional capacity in the children who survived a brain tumor; (2) to explore the relationships between motor outcomes and their potential risk factors, i.e., location of tumor, type of treatment, age at diagnosis, and phase of recovery, and (3) to map the parent-reporting of motor problems during functional daily activities perceived by the parents.

## Materials and methods

### Study design and participants

This study is a cross-sectional study in which children were recruited from the children’s oncology ward of the University Hospitals Leuven. All pediatric brain tumors survivors who were seen for a follow-up consultation in the survivor clinic were invited to participate between 11/12/2017 and 4/11/2019. The study was approved by the local ethical committee of Leuven. Informed consent was obtained from all parents. All children > 12 years gave informed consent themselves as well.

The inclusion criteria were (1) children who were diagnosed with a brain tumor before the age of 16 years old, (2) aged between 3 and 16 years at time of assessment, (3) at least 6 months post-treatment (i.e., neurosurgery and/or biopsy and possible adjuvant therapy), and (4) able to understand and cooperate during the testing procedure. Clinical and demographic data regarding age at diagnosis, tumor location, received treatment, the date of end of therapy, and gender were collected from the medical records (see Table 1).

**Table 1:** Sample characteristics of the participants (*n* = 52)

	Frequency (*n*)	Percentage (%)	6MWT Mean (SD) (*n* = 50)	MABC-2-NL total Mean (SD) (*n* = 49)	BOT-2 fine manual control Mean (SD) (*n* = 44)	BOT-2 body coordination Mean (SD) (*n* = 43)	ABILOCO-Kids Mean (SD) (*n* = 52)	ABILHAND-Kids Mean (SD) (*n* = 52)
Complete dataset			83.20% (14.39)	4.67 (3.84)	41.14 (11.91)	36.81 (11.15)	3.49 (2.36)	4.86 (2.09)
Gender								
Boys	30	57.69	84.9% (12.37)	4.73 (3.54)	40.65 (15.91)	34.00 (10.92)	4.22 (2.07)	4.95 (1.85)
Girls	22	42.31	80.57% (16.99)	4.58 (4.36)	39.33 (10.72)	36.94 (11.16)	3.56 (2.70)	4.72 (2.43)
Diagnosis								
Pilocytair astrocytoma	23	44.23	82.49% (17.24)	5.82 (4.54)	45.65 (13.64)	38.95 (11.51)	3.89 (2.54)	4.85 (2.06)
Medulloblastoma	9	17.31	74.79% (14.00)	1.78 (1.64)	36.25 (5.8)	28.25 (4.74)	2.48 (2.58)	3.70 (2.08)
Other type of tumors	20	38.46	88.02% (8.35)	4.72 (2.95)	37.94 (10.22)	38.53 (25.51)	4.06 (1.75)	5.39 (2.02)
Localization of tumor								
Infratentorial	31	59.62	84.76% (12.76)	4.28 (3.81)	42.54 (9.67)	35.79 (9.19)	4.07 (2.27)	4.81 (2.16)
Supratentorial	21	40.38	81.54% (15.97)	5.25 (3.91)	34.95 (14.23)	38.11 (13.38)	3.76 (2.51)	4.92 (2.05)
Age (years) at time of diagnosis								
< 5 years of age	22	42.31	83.80% (15.35)	5.00 (3.95)	40.65 (15.91)	34.00 (10.92)	3.49 (2.78)	4.22 (2.57)
> 5 years of age	30	57.69	83.21% (13.98)	4.00 (3.81)	41.54 (7.47)	39.04 (11.05)	4.27 (1.97)	5.32 (1.55)
Phase of recovery								
< 2 years	13	25.00	83.81% (14.41)	5.38 (4.57)	42.20 (9.39)	42.60 (14.57)	3.42 (2.59)	3.98 (2.25)
> 2 years	39	75.00	83.03% (14.57)	4.42 (3.57)	40.82 (12.66)	35.06 (9.48)	4.12 (2.28)	5.15 (1.98)
Type of therapy								
Wait and see strategy	2	3.85	82.51% (4.28)	2.00 (1.41)	31.00 (14.14)	29.00 (11.31)	3.13 (0.70)	6.68 (0.00)
Surgery/biopsy	20	38.46	87.85% (13.20)	6.37 (4.11)	47.47 (11.63)	42.35 (12.09)	4.80 (1.67)	5.49 (1.60)
Surgery/biopsy + adjuvant therapy	30	57.69	79.94% (15.02)	3.71 (3.36)	37.64 (10.29)	33.54 (8.98)	3.42 (2.66)	4.31 (2.28)

Test scores involved: 6MWT scores as % reached distance of estimated distance, mean MABC-2-NL standard score, mean BOT-2 standard score, and mean ABILOCO and ABILHAND-Kids questionnaire logits

### Motor assessment

All children were assessed with a test battery during a follow-up consultation at the hospital by a team of three physiotherapists and two physiotherapists in training. The clinical test battery was composed as such to cover multiple aspects of motor function.

First, the Movement Assessment Battery for Children (MABC), second edition, Dutch version (MABC-2-NL) [19] was administered to evaluate manual dexterity, aiming and catching, and balance. The test is designed for children between 3 and 16 years old. Standard scores and percentile scores were derived from the raw scores, using Flemish norm values. The psychometric properties of the first version of the MABC have been well established with an interrater reliability of 0.95–1, which is similar for the second version [19]. A study of Smits-Engelsman showed an excellent test–retest reliability of the MABC-2 [20].

Second, fine motor function, balance, and coordination were further examined using the BOT-2 [21] which consists of four motor domains: fine manual control, manual coordination, body coordination, and strength and agility. For this study, we only included fine manual control (consisting of two subscales fine motor precision and fine motor integration) and body coordination (consisting of subscales balance and bilateral coordination). This is a motor function test for children between 4 and 21 years. Raw data were normalized using American gender-combined norm values. The psychometric properties of the BOT-2 are well established for typically developing children, with a high inter-rater reliability and internal consistency of *r* = 0.79–0.85 and *α* = 0.95–0.96, respectively [21].

Functional walking capacity was assessed with the 6-min walk test (6MWT) [22], measuring the distance a child can walk in 6 min time. This distance was compared to norm values according to Takken et al. [23], taking age, length, and weight into account. The achieved score was compared to this norm and expressed as a percentage of the expected distance.

### Parent-reported motor complaints

Parental perception about fine motor and functional locomotor abilities was assessed using two questionnaires and a standardized anamnesis. The questionnaires evaluate functional locomotor abilities in several functional activities (ABILOCO-Kids) and manual dexterity (ABILHAND-Kids) in daily life of children from 6 to 15 years of age. The completed questionnaires are scored using a Rasch analysis, which results in a logit score [24, 25]. In both questionnaires, parents or adolescents rated the items on a 3-point ordinal scale: impossible, difficult, or easy. The questionnaires have a good test–retest reliability and validity in children with neurological conditions [24, 25]. The anamnesis consisted of standardized open questions focusing on physical complaints, problems with activities of daily living, problems with fine motor skills, and problems with balance (Supplementary information S1).

### Statistical analyses

First, normal distributions of linear variables were checked with Q-Q-plots. After confirmation of the normality assumption, parametric statistical tests were implemented.

For our first objective, patient scores were compared with the test-specific normative values (i.e., mean score of 10 and 50 of the MABC-2-NL and BOT-2, resp., and 100% of the expected distance on the 6MWT), using one-sample T-tests. In addition, frequencies of standard scores and percentile scores were reported. Scoring of the MABC-2-NL components (mean = 10 and SD = 3) results in three descriptive categories: green (pc16 < pc, -1SD < x), orange (Pc6-16, -2SD < x < -1SD), and red zone (pc < pc6, x ≤ -2SD), indicating a “normal” versus “at risk” score and a “need for physiotherapy,” respectively. For the BOT-2, standard scores for the two main domains have a mean of 50 with a SD of 10. These were classified into 5 descriptive categories: well-above average (≥ 2SD), above average (≥ 1SD), average (< 1SD—> 1SD), below average (≤ 1SD), and well-below average (≤ 2SD).

For our second objective, we examined the main effects of brain tumor location (supratentorial/ infratentorial), surgery (yes/no), adjuvant therapy (yes (chemo-and radiotherapy)/no), age at diagnosis, and recovery duration on the outcome, using a stepwise linear regression model for each scale of the motor assessments as outcome. In case of significant main effects, interaction models were additionally tested using ANOVA-tests, which included the significant main predictors.

For our third objective, Pearson correlations were calculated between the logit scores of the ABILHAND-Kids and the ABILOCO-Kids questionnaires and motor test scores. Floor or ceiling effects were considered to be present if more than 15% of respondents achieved the lowest or highest possible score, respectively [26].

All data were analyzed using SPSS v.25 (IBM), with a level of significance of *p* < 0.05. We corrected for multiple comparisons for the one-sample T-tests (*n* = 6) based on the Bonferroni-procedure. Given the exploratory approach for risk factor analyses, the Bonferroni-procedure was not executed for the stepwise linear regression models (*n* = 6).

## Results

### Participants

Out of 65 patients who were seen in the survivor clinic, 52 children were included (30 boys and 22 girls). The mean age at time of assessment was 11.7 years (SD = 43 months) range between 3.3 and 16.8 years. Mean age at time of diagnosis was 6.2 years (SD = 47 months) range between 0 and 13.9 years. The average phase of follow-up since end of therapy was 4.7 years (SD = 36 months) range between 6 months and 10.5 years. Sample characteristics on the type of tumor, type of therapy, age at diagnosis, and phase of recovery are presented in Table 1.

### Description of motor function

Patients performed significantly lower than the normative mean on all subscales (MABC-2-NL manual dexterity, aiming and catching, balance, and total score; BOT-2 fine manual control and body coordination) and reached lower distances on the 6MWT than expected (*p* < 0.001), which remained significant for each subscale after Bonferroni correction (*p* < .001) (Table 2).

**Table 2:** Results of the one-sample T-tests

	*T*-value	df	*p*-value	Mean difference	Lower bound 95% CI	Higher bound 95% CI
MABC-2-NL manual dexterity	−8.04	48	< .001***	−4.27	−5.33	−3.20
MAB2-NL aiming and catching	−8.21	48	< .001***	−4.41	−5.49	−3.33
MABC-2-NL balance	−7.73	48	< .001***	−4.29	−5.40	−3.17
MABC-2-NL total score	−9.72	48	< .001***	−5.33	−6.43	−4.22
BOT-2 fine manual control	−4.94	43	< .001***	−8.86	−12.48	−5.24
BOT-2 body coordination	−7.75	42	< .001***	−13.19	−16.62	−9.75
6MWT	−8.237	49	< .001***	−16.82	−20.92	−21.72

^***^indicates a significant *p*-value at Bonferroni-corrected $\alpha$-level < .001

**Fig. 1:**
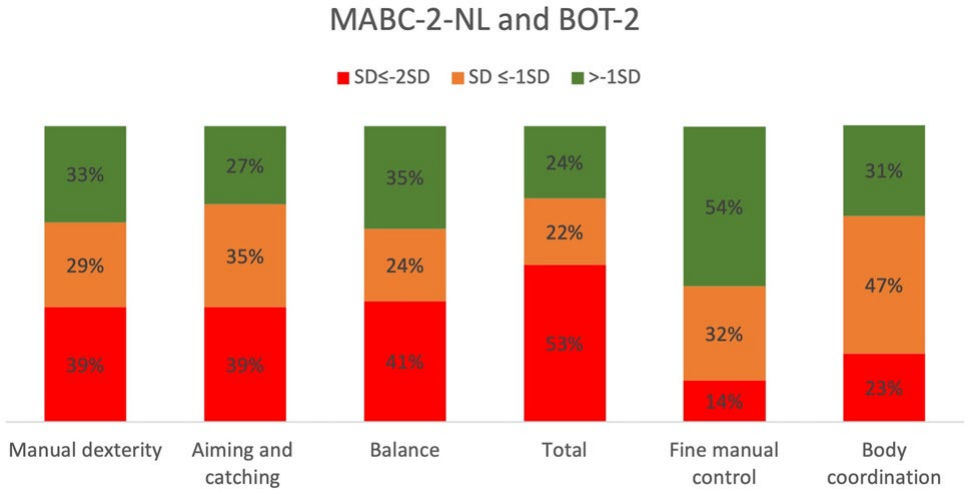
Frequency distribution of the MABC-2-NL different components and total score and BOT-2 on fine manual control and body coordination

Table 1 shows the means and standard deviations of the achieved test scores on each motor assessment. Out of the 52 children, 3 were unable to complete the MABC-2-NL due to practical reasons. On the MABC-2-NL subscales manual dexterity, aiming and catching, balance, and total score, 68%, 64%, 65%, and 75% of the patients scored ≤ -1 SD, respectively (Fig. 1). Regarding the BOT-2, 46% and 70% of the children scored ≤ -1 SD on fine manual control and body coordination, respectively.

**Fig. 2:**
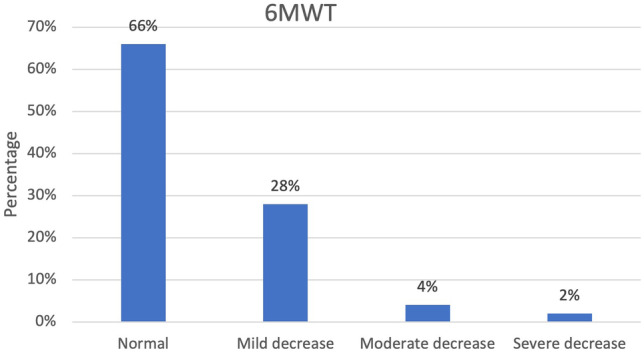
Frequency distribution of the 6MWT

Sixty-six percent of the children scored normal on the 6MWT (> 82% of estimated distance), and 34% scored mildly to severely lower than expected on the 6MWT (Fig. 2) (*p* < .001).

### Stepwise linear regression model of potential risk factors

Lower scores on MABC-2-NL balance (*p* = 0.036) and BOT-2 fine manual control (*p* = 0.024) were associated with adjuvant treatment. A lower score on body coordination BOT-2 (*p* = 0.006) was significantly associated with younger age at diagnosis (< 5 years). All other motor component scores were not significantly predicted by either of these potential risk factors. Based on these results, an additional interaction model was tested including adjuvant treatment, age at diagnosis, and their interaction term, to predict MABC-2-NL balance, BOT-2 fine manual control, and BOT-2 body coordination. None of these interaction effects was significant (Table 3).

**Table 3:** Results of the stepwise linear regression model

Main effects	
MABC-2-NL manual dexterity	No significant predictors
MABC-2-NL aiming and catching	No significant predictors
MABC-2-NL balance	Adjuvant tx (*B* = −.30, *T* = −2.16, *p* = .036)
6MWT	No significant predictors
BOT-2 fine manual control	Adjuvant tx (*B* = −.34, *T* = −2.35 *p* = .024)
BOT-2 body coordination	Age at diagnosis (*B* = .41, *T* = 2.89, *p* = .006*)

*p* indicates the original *p*-value

* indicates a significant *p*-value at $\alpha$-level < .05

### Questionnaire data

**Fig. 3:**
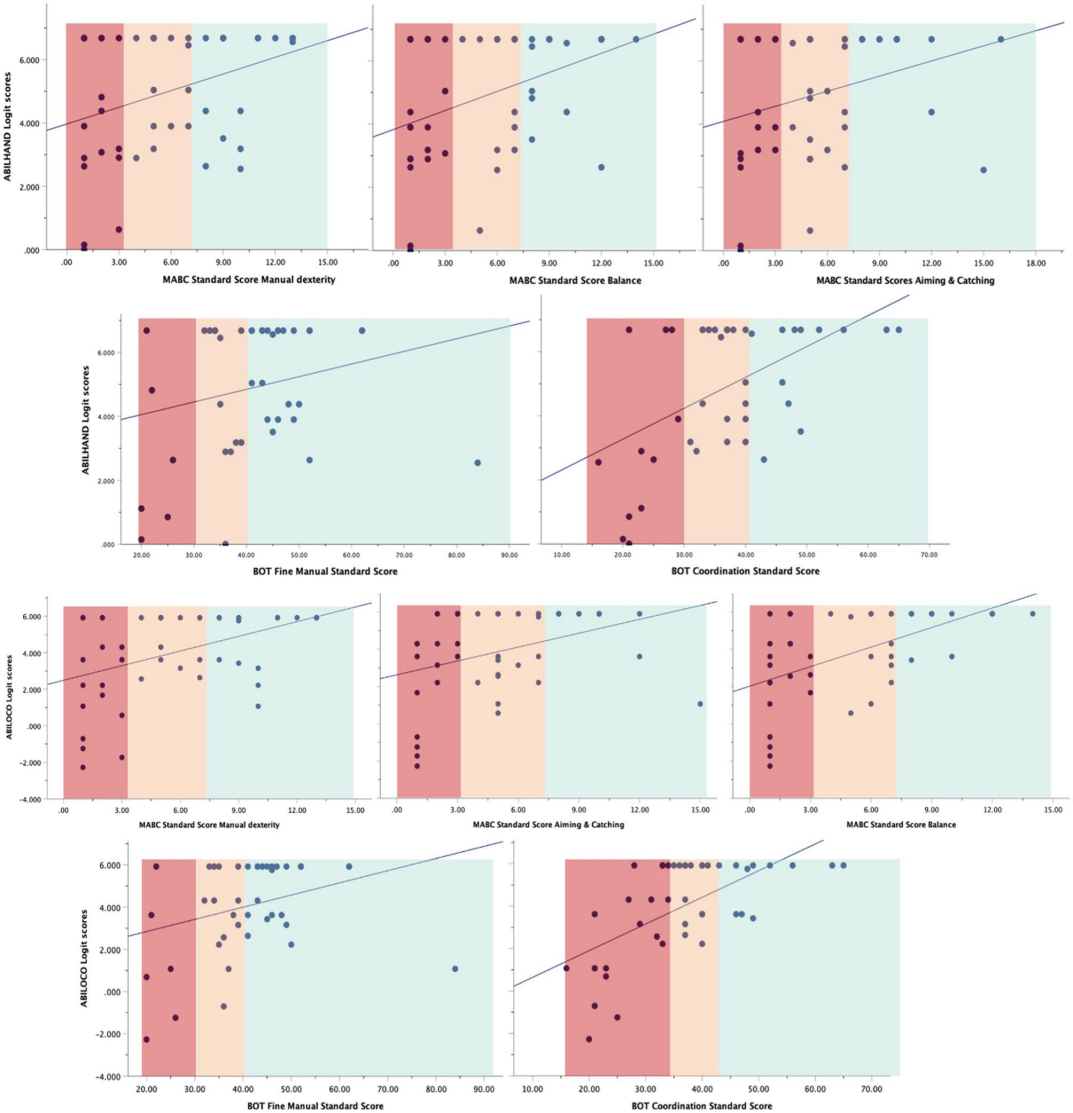
Scatterplots Note. On the y-axis, ABILHAND and ABILOCO Kids questionnaire scores are presented, against scores of the MABC-2-NL manual dexterity, aiming and catching, balance, BOT-2 fine manual control, and body coordination on the x-axis

The group mean logit scores on the questionnaires are presented in Table 1. Based on the ABILOCO-Kids and ABILHAND-Kids questionnaire of the children, respectively 48% and 50% reached maximum scores indicating no physical problems and no problems with daily activities that require the use of the upper limbs. These results indicate a strong ceiling effect (Fig. 3). The Pearson correlation between the ABILHAND-Kids and ABILOCO-Kids was high (*r* = 0.817). Correlations between the questionnaires and the MABC-2-NL scales, BOT-2 fine manual control, and bilateral coordination were varying from weak to good (0.228–0.625) (Supplementary Table S2). Based on the standardized anamnesis, 42% of the parents reported their children to have general physical complaints (back pain, pain in legs, headache, etc.); 39% of them reported that their children have difficulties with independence during activities of daily living (tie laces, closing buttons, wash body, etc.), 59% of them indicated their child to have problems with balance (standing on one leg, walking on the balance beam, etc.); and 40% reported that their child demonstrate problems with fine motor skills (handwriting, drawing, closing buttons, etc.).

## Discussion

This study shows that many children in the follow-up phase after treatment for a brain tumor show impaired motor functioning on multiple aspects (e.g., fine motor skills, balance, and walking distance). Younger age at diagnosis was a significant risk factor for BOT-2 body coordination outcomes, having adjuvant therapy was a risk factor for MABC-2-NL balance and BOT-2 fine manual control. In addition, based on the discrepancies between the questionnaires and anamnesis versus the objective test scores, motor problems appear to be underestimated by the parents.

Across all tests, patients scored significantly lower compared to the normative mean. Based on the total score of the MABC-2-NL, 75% has significant motor function problems indicating a risk of having motor function problems (22%) or need for individualized rehabilitation service (53%). Regarding fine manual control and body coordination (BOT-2), 46% and 70% of the patients scored below average and well below average. Similarly, Macedoni-Luksic et al. [27] indicated that 56% of childhood brain tumor survivors, who were in follow-up for at least 3 years, presented with motor problems. However, Piscione et al.’s study [13] was the only previous study using a standardized objective assessment tool to estimate motor function in children after a brain tumor. They found differences between their cohort and normative data for body coordination and strength and agility of the BOT-2, of which the first scale was similarly reported in our study. Varedi evaluated balance with the sensory organization test in adult survivors of pediatric brain tumors and found balance impairment in 48% of the survivors, which is also in line with our findings [14]. Regarding the 6MWT, 17 out of 50 children walked a significantly shorter distance than their peers, suggesting a reduced functional walking capacity and endurance in childhood brain tumor survivors.

These low scores have an impact on daily life. For example, the anamnesis revealed that these children have a harder time standing on one leg (which impacts getting dressed while standing), walking on the beam in physical education classes, and catching and throwing a ball in activities with peers. Parents also report writing difficulties and that their child is tired faster than peers. The need of rehabilitation interventions should thus be emphasized, in order to reduce the impact on their daily lives, both for functional activities and cardiovascular fitness.

A significant effect was found for age at diagnosis (BOT-2 body coordination), with patients who were diagnosed at younger age (< 5 years) being more at risk. Beuriat et al. [28] also observed degraded motor outcomes (EORTC QLQ-C30 performance scale, International Cooperative Ataxia Rating Scale, Purdue Pegboard Test) in patients who had neurosurgery before 7 years of age for a fossa posterior tumor. By contrast, Piscione et al. [13] found no significant relation between motor function and age at diagnosis or time since diagnosis. However, these studies were performed in infratentorial tumors only.

Time since treatment was not significantly associated with MABC-2-NL, BOT-2, or 6MWT scores. This could be explained by the fact that children reach a plateau. Therefore, they probably need more rehabilitation in daily life to improve the motor function abilities.

No performance differences were found according to the location of the brain tumor. An earlier study (with low-grade astrocytoma patients) reported that significantly more children with supratentorial tumors had impairments [7]. Additionally, the involvement of deep cerebellar nuclei lesions can also have an impact on motor and cognitive functioning [28]. Caeyenberghs et al. [29] revealed that not only the location of the acute injury, but also the secondary white matter damage can highly influence balance in children with traumatic brain injury. Cranial radiation therapy (CRT) in pediatric oncology patients induces significant loss of white matter, as well as decreased gray matter volume [30, 31]. Also surgery and chemotherapy treatment in posterior fossa brain tumor affected the brain microstructure. Biomarkers indicating cellular changes in the thalamus, hippocampus, pons, prefrontal cortex, and white matter tracts were associated with lower psychometric scores [32]. Whether white matter damage in brain tumor survivors could explain motor outcomes could be investigated in future neuroimaging studies. Furthermore, the chemotherapeutic agent vincristine is known to induce peripheral neuropathy. Given that almost all patients who received chemotherapy received vincristine specifically in this study, we cannot exclude the possible neurotoxic mechanisms due to vincristine in this subgroup. Vincristine-induced neuropathy might have led to long-term difficulties in balance and fine motor skills. This research question should be included in future trials.

Questionnaires reached a ceiling effect. Therefore, the full range and possible changes in scores cannot be sufficiently captured, so time-dependent effects are not detected. Furthermore, parent-reported complaints often differ from patient-reported outcomes [7]. The lack of differentiation between reports is a limitation in this study, and it is advised to be included in future research. Based on our anamnesis, we revealed that 58% of parents indicate that their children have no physical complaints and 61% reports no problems with independence during daily living. When interviewing more in depth, with examples of specific forms of motor functioning 59% and 40% of the parents indicate their child to have problems with balance and fine motor skills, respectively. Given the low correlations between the objective motor test battery and questionnaires, parent-report findings suggest a general underestimation of long-term motor problems in this population, especially when questions are not sufficiently specific. Therefore, a systematic assessment of objective motor functioning is highly recommended for this population at international scale. Piscione et al. [33] performed an intervention study in childhood brain tumor survivors. Due to their focus on a physical training program, the largest improvement was obtained on the BOT-2 subscale of bilateral coordination. This study provided first evidence that exercise training may improve the motor function abilities in childhood brain tumor survivors. Also at cerebral level, brain tumor survivors could benefit from a training program, repairing white matter integrity, cortical thickness [34], and improving reaction times [35].

Although the current study is exceptional in reporting objective motor functioning in patients with childhood brain tumor, there are several limitations in this study. First, the test battery was not specifically validated in this population. However, to date, there are no previously validated assessment tools for motor function abilities in childhood brain tumor survivors specifically. For this reason, we composed a custom-made clinical test battery to cover several aspects of motor function and daily life activities that are clinically relevant in this patient group. In particular we aimed to assess gross and fine motor function, balance, functional mobility, as well as the capabilities in functional activities of daily living. As no specific measures for children with brain tumors are currently recommended at international scale, a literature search was performed to identify available tests and parent questionnaires used in children diagnosed with neurological and other developmental disorders. The tests and questionnaires that were reported in the literature were discussed by an expert group of specialized physiotherapists in pediatric oncology, a pediatric neuro-oncologist, and involved researchers in pediatric rehabilitation. The selection process was based on the following criteria: content of the tests and questionnaires relevant for children with brain tumors, standardized, age-appropriate assessment tools with good reliability and validity, the availability of norm values, limited test duration, and efficient assessment, with a maximal assessment time of 45 min to 1 h, to ensure compliance of the child.

Further assessment of the reliability and validity of the already existing tools in this population is therefore warranted. Another limitation is the fact that we did not measure cognitive domains such as visual functioning, attention, or executive functioning, which may have influenced motor coordination performance.

In this study, children were consecutively recruited at follow-up consultation timepoints. The results indicate that the composed test battery does identify the children with motor function problems, who would not be detected in routine medical follow-up due to more mild problems. This study is therefore a first step towards understanding the motor function abilities in brain tumor survivors. These results emphasize the need for good screening instruments for motor function abilities (coordination, balance, and fine motor skills) and adequate rehabilitation programs.

Concludingly, longitudinal follow-up with standardized assessments is recommended to gain full insight into the motor functioning of childhood brain tumor survivors, which is needed for future therapy recommendations for these children.

## Supplementary Information

Below is the link to the electronic supplementary material.

Supplementary file1 (DOCX 14 KB)

## Data Availability

Anonymized data are available upon request.

## Abbreviations

6MWT: 6-Min walk test
ABILOCO-Kids: Locomotion ability measure for children
ABILHAND-Kids: Manual ability measure
ANOVA: Analysis of variance
BOT-2: Bruininks-Oseretsky test of motor proficiency
HRQL: Health-related quality of life
MABC-2-NL: Movement Assessment Battery for Children, second edition, Dutch version
SD: Standard deviation
SPSS: Statistical Package for the Social Sciences
