# Correction to: Better detection of reduced motor functioning in brain tumor survivors based on objective motor assessments: an incentive for improved standardized follow-up

**DOI:** 10.1007/s00431-022-04605-6

**Published:** 2022-09-08

**Authors:** Marjoke Gielis, Veerle Dirix, Ellen Vanderhenst, Anne Uyttebroeck, Hilde Feys, Charlotte Sleurs, Sandra Jacobs

**Affiliations:** 1 Department of Pediatrics, Pediatric Hemato-Oncology, University Hospitals Leuven, Herestraat 49, Leuven, Belgium; 2 Department of Oncology, KU Leuven, Leuven, Belgium; 3 Department of Rehabilitation Sciences, KU Leuven, Leuven, Belgium

## Correction to: European Journal of Pediatrics (2022) 181:2731–2740 https://doi.org/10.1007/s00431-022-04472-1

In the original published version of the above article, the authors’ names were incorrectly presented. The names are now corrected and cited correctly as, “Gielis, M., Dirix, V., Vanderhenst, E., Uyttebroeck, A., Feys, H., Sleurs, C., Jacobs, S.”

The original article has been corrected.

