# Supplementary material for: Better detection of reduced motor functioning in brain tumor survivors based on objective motor assessments: an incentive for improved standardized follow-up
Source: Eur J Pediatr. 2022 Apr 27;181(7):2731–40. doi: 10.1007/s00431-022-04472-1 (PMC9192471; doi:10.1007/s00431-022-04472-1)
Supplement: Supplementary file 1 — Supplementary file1 (DOCX 14 KB) [file 431_2022_4472_MOESM1_ESM.docx]

Supplementary information

SUPPLEMENTARY S1: STANDARDIZED ANAMNESIS

1. Do you currently have any physical symptoms? (YES/NO)
   1. Specify (what symptoms, when do you suffer from them, are you unable to do things because of them?
2. Do you notice any problems with balance? (YES/NO)
   1. Specify
3. Do you notice any problems with fine motor skills? (YES/NO)
   1. Specify
4. Can you do everything independently (get dressed, tie laces,...)? (YES/NO)
   1. Specify (what does not work independently)
5. Do you participate in sports?
   1. Specify (what sport, how often?)
6. Do you have any other hobbies?
   1. Specify (what hobbies, how often?)
7. Can you participate in everything during gymnastics/LO classes at school?
   1. Specify
8. Do you or have you ever received physical therapy?
   1. Specify (when, how long, how often?)

SUPPLEMENTARY TABLE S2 Pearson correlations between questionnaires and motor test assessments

|  | ABILOCO-Kids | ABILHAND-Kids |
| --- | --- | --- |
| ABILHAND-Kids | 0.82* | 1 |
| ABILOCO-Kids | 1 | 0.82** |
| MABC-2-NL Manual Dexterity | 0.43** | 0.33* |
| MABC-2-NL Aiming & Catching | 0.40** | 0.30* |
| MABC-2-NL Balance | 0.58** | 0.39** |
| MABC-2-NL Total | 0.45** | 0.33* |
| BOT-2 Fine Manual Control | 0.31* | 0.23 |
| BOT-2 Bilateral Coordination | 0.63** | 0.51** |

**Correlation is significant at the level 0.01 (2-tailed), * Correlation is significant at the level 0.05 (2-tailed).
